# The punched-out tissue complex (skin-bone ”imprimatum“) in shots from captive-bolt guns: does it act as a secondary projectile?

**DOI:** 10.1007/s00414-020-02262-8

**Published:** 2020-02-14

**Authors:** Dorothee Geisenberger, Arianna Giorgetti, Matthieu Glardon, Markus Große Perdekamp, Stefan Pollak, Rebecca Pircher

**Affiliations:** 1 Institute of Forensic Medicine, Faculty of Medicine, University of Freiburg, Albertstraße 9, 79104 Freiburg, Germany; 2 Department of Legal and Occupational Medicine, Toxicology and Public Health, University of Padova, Via Falloppio 50, 35121 Padova, Italy; 3 Center of Forensic Physics and Ballistics, Institute of Legal Medicine, University of Berne, Bühlstrasse 20, 3012 Berne, Switzerland

**Keywords:** Captive-bolt stunning device, Slaughterer’s gun, Skin-bone imprimatum, Secondary projectile, Ballistic simulants

## Abstract

From the first half of the twentieth century to the present day, injuries and fatalities from captive-bolt livestock stunners are a major topic in forensic medicine. The vast majority of cases account for suicides with the frontal, temporal, parietal, and occipital regions being the most common sites of entrance (in descending order of frequency). Due to the limited length of the bolt, the corresponding wound channel within the braincase is only several centimeters long. It has been a controversial subject for a long time, whether the skin-bone complex punched out by the conically grooved end of the steel rod may act as a “secondary projectile” being propelled beyond the actual path of the bolt. To answer this question, experimental shots from various types of captive bolt-guns were fired to simulants. Video-documentation employing a high-speed motion camera showed that the punched-out pieces of skin and bone did not move further than the bolt. Thus, a secondary extension of the total wound channel could not be observed. However, the suction effect caused by the bolt’s rearward movement may induce a slight retrograde displacement of the skin-bone complex.

## Introduction

Powder-activated captive-bolt livestock stunners are still of great forensic importance, especially in the central European countries [43, 45]. Such devices are used to cause immediate unconsciousness in animals to be slaughtered. They are composed of a cylindrical metal tube containing an inlying steel rod, the so-called bolt, which is propelled by the powder gases of a blank cartridge [12]. The strength of the ammunition is indicated by color markings on the base of the cartridges. The bolt has a diameter of about 10 mm, a circular cross-section, and a conically grooved end acting as a sharp-edged punching tool when driven out from the gun’s muzzle with an initial velocity of about 50 m/s or less [9, 36]. The penetration depth depends on the bolt’s length and usually does not exceed 10 cm. After firing, bolts mostly return into the barrel by a rubber bushing and/or by a withdrawal spring although some models lack a return mechanism and must be drawn back by hand [42]. Several makes of captive-bolt stunners have two or four openings for the combustion gases. They are typically located in the muzzle plane beside the central hole for the bolt and determine characteristic smoke soiling patterns in contact and close range shots [6, 25, 31, 36, 40, 44, 45].

At the entrance site of the bolt, its grooved end produces a sharp-edged punch lesion of the skin and a circular hole of the underlying (flat) bone. The hole in the outer table of the cranial vault roughly corresponds to the diameter of the bolt whereas the inner table is broadened in a crater-like manner. The skin-bone complex (possibly accompanied by any head covering and/or scalp hair) is impacted at the front end of the bolt and carried along into the cranial cavity. If the bacterially contaminated skin is deposited in the depth of the brain, inflammatory sequelae are to be expected in cases of primary survival [4, 5, 10, 15, 41, 56].

Ever since the forties of the last century, some authors maintained that the distance between the entrance wound and the final position of the imprimatum may be longer than the bolt’s penetration depth [4, 18, 25, 34]. In this context, the imprimatum was labeled as a “secondary projectile” [4, 14, 25, 31, 34, 46] responsible for a lengthening of the wound channel beyond the way traveled by the bolt. In the context of gunshot wounds from conventional firearms, the conception of bone fragments acting as secondary missiles is met with skepticism [28, 49].

To answer the question, whether the skin-bone imprimatum moves further as well after the bolt has finished its forward motion, test shots with various types of captive-bolt guns were fired to simulants using video-documentation with a high-speed camera.

## Material and methods

For the test shots, 2 composite models (a 2- and a 3-component model) as well as 3 different captive-bolt guns were used.

**Fig. 1:**
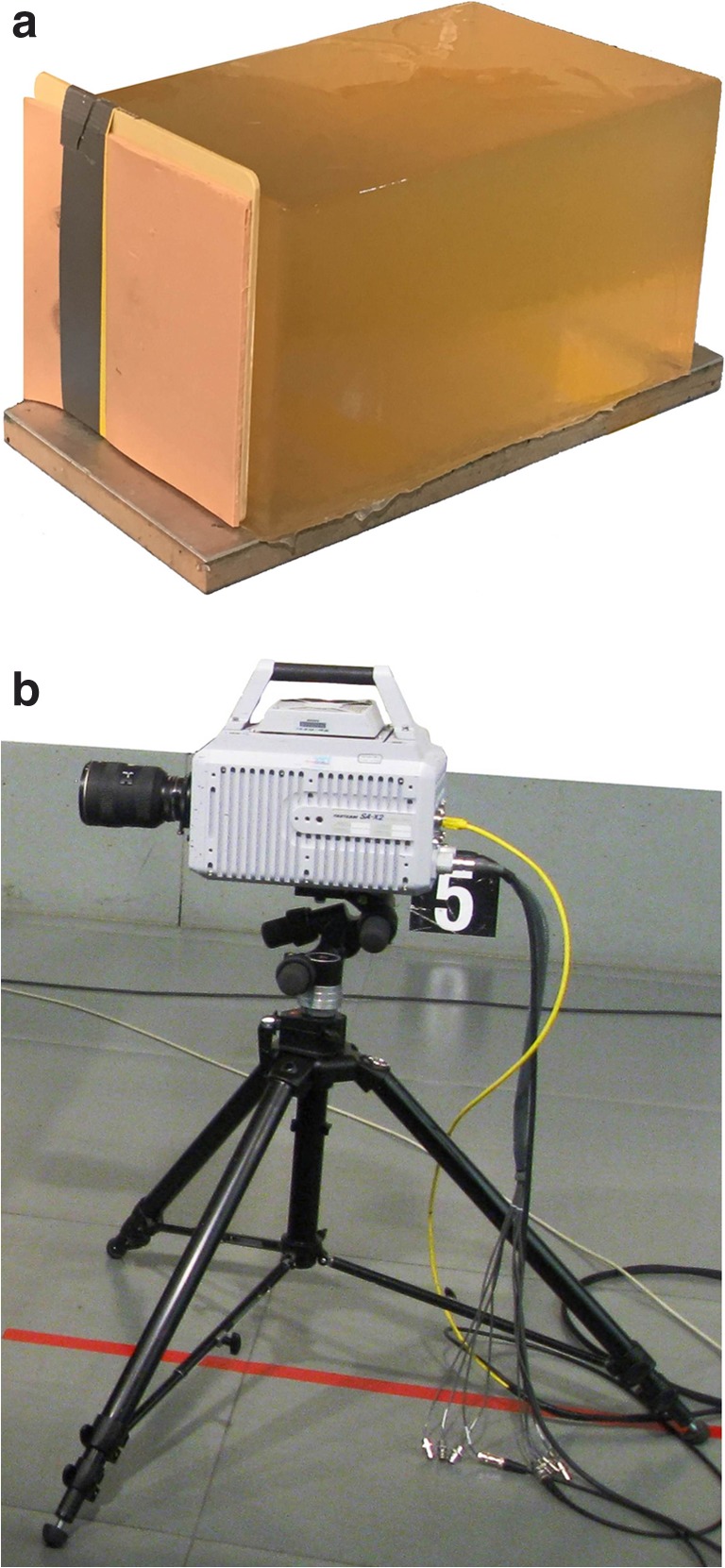
**a** 3-component model consisting of artificial skin and a synthetic bone plate in front of a gelatin block. **b** High-speed-camera (Photron Limited, Fastcam SA-X2, Tokyo, Japan)

The 2-component composite model consisted of a synthetic bone plate and a gelatin block as soft-tissue simulant. The 3-component model was composed of artificial skin, synthetic bone, and gelatin (Fig. 1).

As a skin simulant, a 5-mm thick, 2-layer synthetic material (Ecoflex 0030, Synbone®, Zizers, Switzerland) was used, which has elastic properties similar to human skin. The plate-shaped 3-layer bone simulant made from polyurethane (Synbone®, Zizers, Switzerland) was 6 mm thick in all. The gelatin blocks were 25 × 25 × 40 cm in size; they were prepared from a 10% solution (250 Bloom, type A, grain 20/60) according to current recommendations for experiments in wound ballistics.

**Fig. 2:**
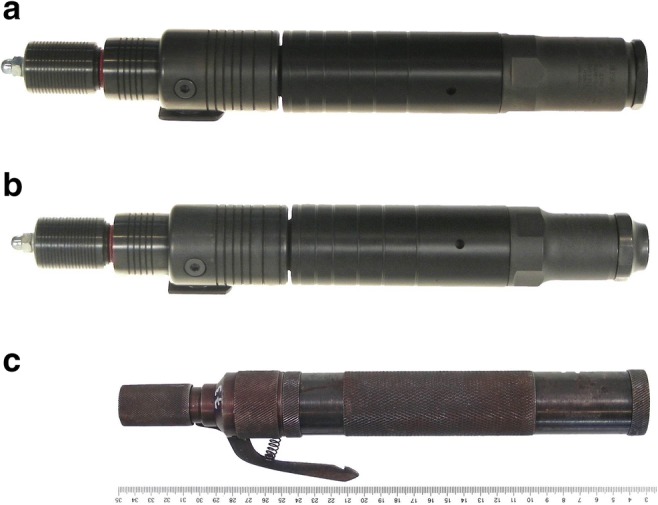
The captive-bolt guns used for the test shots. **a** Make Schermer KR. **b** Make Schermer KS. **c** Make Pfeiffer

The following captive-bolt guns were used (Fig. 2):

**Table:** 

Manufacturer	Type	Cartridge caliber	Bolt length	Bolt diameter	Bolt velocity
Schermer	KR	6.8 × 15 mm	8.5 cm	12 mm	45–65 m/s
Schermer	KS	6.8 × 15 mm	8.0–8.5 cm	12 mm	45–65 m/s
Pfeiffer	–	9 × 17 mm	6.0 cm	13 mm	Not indicated

With the “Schermer KR“ gun, the bolt does not automatically return to its original position after firing the shot, whereas the captive-bolt models “Schermer KS“ and “Pfeiffer“ are equipped with a bolt retractor system.

The shots with the narcotic devices were fired using either “Schermer” cartridges cal. 6.8 × 15 mm (Schermer GmbH, Ettlingen, Germany) or “Dynamit Nobel“ cartridges cal. 9 × 17 mm (Fürth, Germany), both with a red color marking, which is the most powerful cattle cartridge type (“for heavy bulls“).

With each of the 3 livestock stunners, the following test shots were fired:

- 1 dry-fire,
- 1 contact shot to the 2-component composite model (synthetic bone/gelatin block), and
- 1 contact shot to the 3-component composite model (artificial skin/synthetic bone/gelatin block).

The 9 test shots fired in all were filmed with a high-speed camera (Photron Limited, Fastcam SA-X2, Tokyo, Japan) with documentation at a rate of 5000 frames per second (Fig. 1b).

## Results

### Dry-fire

#### Schermer KR

After firing the captive-bolt gun, the bolt was rapidly driven out from the barrel as far as it would go. At the same time, a dense cloud of gunsmoke with separate powder grains exited from the muzzle before the bolt returned completely to its original position with a subsequent slower full forward movement. This was again accompanied by a smaller cloud of gunsmoke. After a short backward movement of some millimeters, the bolt remained in its resting/final position outside the barrel.

#### Schermer KS

After pulling the trigger, the bolt was rapidly driven out from the barrel as far as it would go followed by immediate retraction to its original position. After another slower forward movement of the stunning bolt (not quite to its maximum length), it was retracted until just before the muzzle and completely returned to its original position after a short delay. There was no visible cloud of gunsmoke.

#### Pfeiffer

After pulling the trigger, the stunning bolt exited through the hole in the muzzle end together with a dense cloud of gunsmoke. Then it was retracted into the barrel approximately half-way and after a short delay finally returned completely to its initial position.

### Shots to the 2-component model (synthetic bone/gelatin block)

#### Schermer KR

From the flat synthetic bone simulant, a round, 12-mm wide and 6-mm-thick fragment was punched out and displaced to the end of the 9.5-cm long penetration channel. In the video sequence, no secondary forward or backward movement of the imprimatum was observed. As after the dry-fire shot, the bolt returned into the barrel and—after another forward movement—reached its final position just behind the imprimatum. In the first section of the channel, for a short time, the maximum diameter was slightly wider than the cross-section of the bolt. In the permanent channel, some small bone particles were found away from the main imprimatum. In the first section of the channel, there were deposits of gunpowder residues visible to the naked eye.

The hole in the synthetic bone plate showed cone-shaped widening in the direction of the shot. The outer edge of the entrance hole was covered with ring-shaped gunsmoke deposits.

#### Schermer KS

**Fig. 3:**
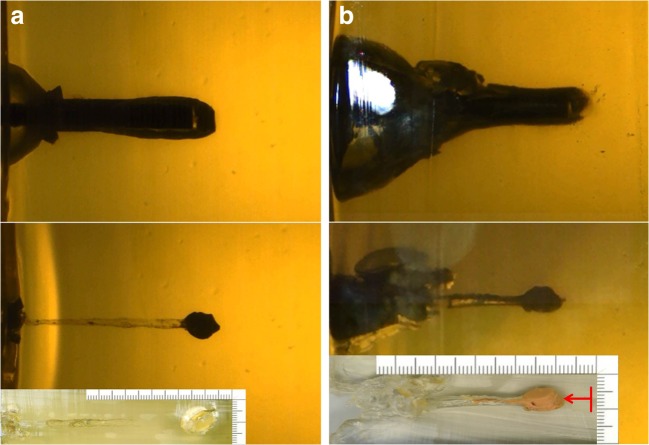
**a** Experimental shot with a captive-bolt gun make Schermer KS to the 2-component model. Lateral view of the gelatin block at the moment when the bolt has reached its maximum penetration depth (top); position of the punched-out complex after complete retraction of the bolt (middle); specimen excised from the gelatin block after the shot showing the permanent bolt channel in gelatin and the imprimatum at its terminal point (bottom). **b** Experimental shot to the 3-component model with a captive-bolt gun make Schermer KS

From the synthetic bone simulant, a round, 13-mm wide and 6-mm-thick fragment was punched out and transported by the bolt to the end of the 8.9-cm long channel. The high-speed photographs showed that a cone-shaped temporary cavity developed only in the initial part of the bolt track and that the secondary forward movement of the bolt did not cause any subsequent displacement of the imprimatum (Fig. 3a). Some small bone fragments had lodged in the initial section of the channel. Gunsmoke residues along the bolt channel were not visible to the naked eye.

At the entrance site, the bone plate showed a vague cone-shaped extension in the direction of the shot. In the zone surrounding the entrance hole, minor soot blackening was seen on the outer margin.

#### Pfeiffer

The findings were similar to those of the Schermer KS device. Again the bolt punched out a fragment of synthetic bone with a maximum diameter of 13 mm and displaced it to the end of the 6.2-cm long bolt track. After the forward movement of the bolt, the imprimatum did not move either forward (i.e., in the direction of the shot) or backward.

**Fig. 4:**
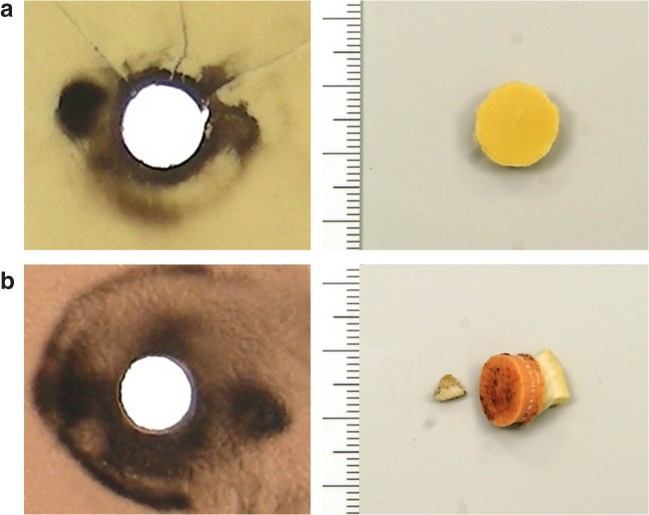
**a** Entrance site in bone (2-component model, contact shot fired with a gun make Pfeiffer) on the left side and punched out bone fragment on the right side. **b** Entrance site in skin (3-component model, contact shot fired with a gun make Pfeiffer) on the left side and punched out skin-bone fragment on the right side

The margin of the entrance hole in the bone plate showed ring-shaped smoke soiling (Fig. 4a) and fracture lines radiating away from the entrance hole. The inner table of the flat bone simulant was perforated in a crater-like manner.

### Shots to the 3-component model (artificial skin/synthetic bone/gelatin block)

#### Schermer KR

From the artificial skin, a round, 5-mm thick piece with a maximum diameter of 11 mm and two adhering bone fragments with a size of up to 8 × 6 mm was punched out. This skin-bone complex was displaced by the penetrating bolt to the end of the 8.6-cm long bolt track. The high-speed video-documentation showed a brief cone-shaped widening of the channel in the initial section and tapering in the distal section. The entire length of the bolt channel was blackened by soot; it contained several bone fragments, especially near the entrance zone.

On the outer margin of the entrance hole in the artificial skin, there was an up to 8-mm wide ring of soot soiling. The synthetic bone showed a 12-mm wide circular defect with cone-shaped widening in the direction of the shot.

#### Schermer KS

From the artificial skin, a 5-mm-thick and 10-mm-wide piece was punched out and displaced to the end of the 7.2-cm long bolt track. The synthetic bone fragment had separated from the covering skin. As the bolt retracted, the imprimatum moved backward (in the direction of the entrance) for 8 mm (Fig. 3b). The channel showed temporary expansion near the entrance and contained several fragments of synthetic bone up to 8 mm in size.

At the margin surrounding the entrance hole, only minor soot soiling was discernible. The bone defect showed a slight cone-shaped widening in the direction of the shot with radiating fracture lines.

#### Pfeiffer

From the artificial skin, an 11-mm-wide and 5-mm-thick cylindrical piece with a synthetic bone fragment adhering to it was punched out and displaced to the end of the 6.6-cm-long channel. After the bolt had traveled to its maximum penetration depth, the skin-bone complex did not move any more. The whole bolt track was blackened by gunsmoke.

The zone around the entrance hole showed intense soot soiling over a width of up to 12 mm (Fig. 4b) and the underlying bone was shattered into small fragments. After removing the bone plate, the fragments were found adhering to the outer side of the gelatin block.

## Discussion

The medico-legal literature is abundant of case reports and reviews concerning fatalities from captive-bolt stunners. Most of them (about 85%) are suicides committed by males experienced in slaughtering procedures [3, 22, 29, 32, 33, 38, 45, 60]. Besides, a remarkable number of homicides [2, 7, 13, 20–22, 24, 27, 29, 32, 38, 40, 46, 48, 51, 53, 55, 61] and several accidents (survived or with fatal outcome) [22, 26, 38, 41, 54, 56, 62] have been published.

In some suicides, apart from the slaughterer’s gun, another suicide method such as hanging is applied [18, 23, 29, 31, 32, 35, 37, 38, 40, 46, 51, 59, 62]. Exceptionally, even shots to the neurocranium need not cause immediate unconsciousness so that the victim may be capable of reloading the device and firing a second shot [1, 11, 16, 38, 40, 50, 57, 62]. Furthermore, two-gun suicides committed by shots from different livestock stunners have been reported [30, 39]. In slaughterer’s guns supplied with rubber bushings and/or recoil springs, the removal of these constituents prior to firing [31, 34, 42] can lengthen the penetration depth and even cause the bolt to break so that it becomes a real projectile [42]. If the bolt does not rebound into the barrel, it gets stuck in the skull [31, 32, 34, 35, 45, 46, 54, 59].

Up to the nineties of the last century, it was thought, that shots to the head inflicted with a slaughterer’s gun do not involve an abrupt increase in intracranial pressure followed by indirect fractures or cortical contusions. Rabl and Sigrist [47] were the first to describe skull fractures away from the bolt’s track. Later on it was shown, that indirect fractures may occur due to a hydraulic burst effect even in shots from captive bolt stunners [17, 18]. Another fundamental and still open question is related to the punched out skin-bone complex which has been regarded as a secondary projectile by some authors [4, 14, 25, 31, 34, 46].

In conventional gunshot injuries to bones, the energy transferred to osseous fragments is considered insufficient to produce separate wound channels. The splinters usually do not precede but follow the bullet as it passes through the soft tissues. Due to the development of a temporary cavity and the negative pressure in it, the bone fragments also move laterally before coming to rest, so that there may be some distance from the permanent wound channel [28, 49]. A final position far from the bullet’s track and embedded in soft tissue may suggest a propulsive movement of the splinter itself. Based on these considerations and confirmed by high-speed images, the term “secondary projectile” does not appear to be appropriate—at least in gunshot injuries from firearm bullets. Nevertheless, the opinions of authors are inconsistent even in this respect. So the monograph of Di Maio [8] contains the following statement: “Sometimes the bone chips create secondary tracks that deviate from the main path.”

Injuries from captive-bolt guns are comparable with those from conventional firearms to a minor degree only. The main differences concern the initial velocity of bolts in relation to bullets, the shape of the bolt’s anterior end (conically grooved, sharp-edged) versus common bullet configurations (round nose, hollow-point, wadcutter, etc.), as well as the bolt’s retraction movement after having reached its maximum penetration depth. Furthermore, the bolt acts like a punching tool as the sharp edge of its front cuts out a corresponding piece of the scalp. The underlying flat bone is broken out in a characteristic manner: The circular hole in the outer table of the skull has a clear-cut edge and roughly complies with the diameter of the bolt, whereas the inner table is beveled resulting in a funnel-like appearance. The punched-out pieces of skin and bone (the latter one mostly fragmented) often retain their anatomical connection so that they are jointly displaced into the depth. Only in very few cases (especially in acute-angled shots), the cut-out skin flap may remain linked to the edge of the entrance hole implicating that the “imprimatum” lacks a dermal component [10, 35, 40, 41, 44, 51].

After being punched out, the skin-bone complex gets embedded in the excavated end of the bolt. There the so-called “imprimatum” is accelerated up to the bolt’s velocity. When the bolt has completed its forward motion, the impacted skin-bone complex separates from the rod. A significant onward movement in the previous direction could not be observed in our experiments. Obviously, the kinetic energy of the “imprimatum” is not sufficient to overcome the penetration resistance of the simulant. The cross-sectional area of the skin-bone complex is large resulting in a minor sectional density considering the comparatively low mass of the punched-out tissue.

When discussing any additional wound lengthening produced by the “imprimatum,” one has to take into account that a large-sized skin-bone-complex may protrude beyond the bolt’s excavated end. Therefore, a minor extension of the wound path in order of the imprimatum’s thickness does not prove a further onward movement in the sense of a secondary projectile. Under the terms of our test shots, the punched-out skin-bone imprimatum did *not* exceed the penetration depth of the bolt.

As described in the methodical section, the test shots were fired at composite models simulating the scalp, the underlying flat bone of the skull, and the brain. The bolt’s path terminated in a block of translucent gelatin permitting a detailed video-documentation of the motion sequences inside. The experimental design did not completely correspond to the situation in shots to the head as there was no firm encasement comparable with the bony skull. Therefore, the lateral and forward acceleration of the simulant induced by the penetrating bolt was not limited as it is to be expected in a human brain case. Nevertheless, this variation would not inhibit any forward movement of the skin-bone complex exceeding the bolt’s penetration depth. Accordingly, it seems justified to deduce from our test results that the skin-bone plug carried along by the bolt’s end does *not* act as a secondary projectile.

Mostly, the impacted bone is fragmented into pieces when it is punched out by the bolt’s anterior end. This explains that more than one osseous splinter may be present in the cranial cavity of victims injured with a livestock stunner. The term “bone imprimatum” in the proper sense only refers to that piece of the cranial vault which is wedged in front of the conically grooved bolt and displaced right up to the end of its track. Additional bone splinters may be located alongside the wound channel, sometimes just behind the entrance site [4, 5, 58, 59].

In our test series, different models of the Schermer and Kerner type slaughterer’s gun were used. The Schermer type is characterized by the absence of smoke outlets at the barrel’s muzzle end [19, 36]. In contrast, makes of the Kerner type have two opposite outlets for the combustion gases causing paired soot depositions on either side of the entrance hole. Even in contact shots, only a small part of the combustion gases is propelled into the depth of the bolt’s track [17, 52]. Nevertheless, in one of the test shots, an intense soot deposition was found in the initial section of the wound track (a similar observation was reported by Hagemeier et al. [19]).

Contrary to previous perceptions, it is known for several years that shots from slaughterer’s guns may be associated with a moderate radial displacement of the target medium. Accordingly, the first section of the bolt track is widened in a non-elastic simulant (ballistic soap) [17] and accompanied by short cracks in gelatine [17, 52]. The increase in intracranial pressure may result in a hydraulic burst effect causing secondary fractures in thin parts of the skull [17, 18, 47].

The high-speed video-documentation impressively visualizes the dynamic interaction between the bolt, the target medium (gelatine), and the skin-bone complex carried along up to its final position. Albeit far less marked than in conventional gunshots, there was a temporary widening of the bolt’s path. When the bolt moved rearward, the distal part of the track considerably reduced in diameter, obviously due to a transient negative pressure. This observation is in accordance with the findings in ballistic soap as previously reported [17] and also explains a potential retrograde shift of the skin-bone complex which was already described by Janssen and Stieger [25].

## Conclusions

- In head shots from slaughterer’s guns, the impacted skin and bone are punched out by the grooved steel bolt and forced into the depth of the brain, where the dislocated tissue complex can be found at the end of the wound track.
- In the past, several authors held the opinion that the so-called imprimatum acts as a “secondary projectile” potentially lengthening the wound channel.
- In order to verify these statements, test shots were fired to composite models and video-documented by a high-speed camera. The results confirmed that the imprimatum actually does *not* move beyond the penetration depth of the bolt.

## References

[1] Alberton F, Castellani G (1991). Un caso di suicidio per duplice colpo di pistola da macellazione. Minerva Med.

[2] Betz P, Pankratz H, Penning R, Eisenmenger W (1993). Homicide with a captive bolt pistol. Am J Forensic Med Pathol.

[3] Bohnert M, Schmidt U, Große Perdekamp M, Pollak S (2002). Diagnosis of a captive-bolt injury in a skull extremely destroyed by fire. Forensic Sci Int.

[4] Bula-Sternberg J, Laniado M, Kittner T, Bonnaire F, Lein T, Bula P (2011). CT-findings in penetrating captive bolt injuries to the head and brain: analysis of the trauma-related CT-findings and review of the literature. Fortschr Roentgenstr.

[5] Crevenna R, Homann CN, Ivanic G, Klintschar M (1999). Unusual treatment of slaughterer’s gun injury. Injury.

[6] Czursiedel H (1937). Ein Selbstmord mittels eines Bolzenschußgerätes. Dtsch Z Ges Gerichtl Med.

[7] Danek E (1965). Schlachtschussapparat als Mordwaffe. Kriminalistik.

[8] Di Maio VJM (1999) Gunshot wounds. Practical aspects of firearm, ballistics, and forensic techniques, 2nd edn. CRC, Boca Raton pp 262–263, 284–285

[9] Dörfler K, Troeger K, Lücker E, Schönekeß H, Frank M (2014). Determination of impact parameters and efficiency of 6.8/15 caliber captive bolt guns. Int J Legal Med.

[10] Faller-Marquardt M (1991) Analysis of gunshot injuries with a livestock gun. Beitr. Gerichtl Med 49:193–200 1811499

[11] Fanton L, Karger B (2012). Suicide with two shots to the head inflicted by a captive-bolt gun. J Forensic Legal Med.

[12] Frank M, Franke E, Philipp KP, Bockholdt B, Ekkernkamp A (2009). Ballistic parameters of cal. 9 mm x 17 mm industrial blank cartridges (cattle cartridges). Forensic Sci Int.

[13] Fritz E (1942). Merkwürdiger Befund nach Tötung eines Menschen mittels eines Bolzenschuß-Tiertötungsapparates. Arch Kriminol.

[14] Gerlach J (1955). Über Bolzenschußverletzungen des Gehirns. Zbl Neurochir.

[15] Gnjidić Z, Kubat M, Malenica M, Sajko T, Radió I, Rumboldt Z (2002). Epidemiological, forensic, clinical and imaging characteristics of head injuries acquired in the suicide attempt with captive bolt gun. Acta Neurochir.

[16] Grellner W, Buhmann D, Wilske J (2000) Suicide by double bolt gunshot wound to the head: case report and review of the literature. Arch Kriminol 205:162–168 10923170

[17] Große Perdekamp M, Kneubuehl BP, Ishikawa T, Nadjem H, Kromeier J, Pollak S, Thierauf A (2010). Secondary skull fractures in head wounds inflicted by captive bolt guns: autopsy findings and experimental simulation. Int J Legal Med.

[18] Große Perdekamp M, Pircher R, Geisenberger D, Pollak S (2018). Complex suicide committed by a captive-bolt slaughterer’s gun fired to the head and consecutive hanging with unusual findings regarding both methods applied. Arch Kriminol.

[19] Hagemeier L, Greschus S, Schild H, Madea B, Schyma C (2009). Bolzenschussgeräte mit und ohne separate Schmauchkanäle. Rechtsmedizin.

[20] Hardt-Madsen M, Simonsen J (1983). Firearm fatalities in Denmark 1970–1979. Forensic Sci Int.

[21] Hegglin O (1957). Über Tötung und Selbstmord durch Bolzenschußapparat.

[22] Heil K, Schulz S, Morgenthal S (2016). Todesfälle nach Bolzenschuss in den Regionen Leipzig und Chemnitz 1980–2015. Rechtsmedizin.

[23] Hofmann V, Herber F (1984) Über kombinierte und protrahierte Suizide. Kriminal Forens Wiss 53/54:83–88

[24] Isfort A (1961). Bolzenschußverletzungen. Dtsch Z Ges Gerichtl Med.

[25] Janssen W, Stieger W (1964) Verletzungen durch Bolzenschuß-Apparate unter besonderer Berücksichtigung der Spurenmerkmale. Arch Kriminol 134: 26–37, 96–102

[26] Kattimani RP, Shetty S, Mirza H (2016). Accidental bolt gun injury to femur – a case report. J Orthop Case Rep.

[27] Kijewski H, Kampmann H (2000) Criminal and suicidal death with bolt gunshot weapons. An experimental and case contribution. Arch Kriminol 206:150–159 11213447

[28] Kneubuehl BP (2011) Patterns in bullet wounds to bones. In: Kneubuehl BP, Coupland RM, Rothschild MA, Thali MJ: Wound ballistics. Basics and applications. Springer, Berlin Heidelberg, pp 131–133

[29] Koops E, Püschel K, Kleiber M, Janssen W, Möller G (1987). Deaths caused by so-called bolt-setting guns. Beitr Gerichtl Med.

[30] Lemke R (1988) Cited by Lignitz et al [32]

[31] Liebegott G (1948/49) Seltener kombinierter Selbstmord und seine versicherungsrechtliche Auswirkung. Dtsch Z Ges Gerichtl Med 39:351–355

[32] Lignitz E, Koops E, Püschel K (1988). Death caused by projectile guns – a retrospective analysis of 34 cases in Berlin and Hamburg. Arch Kriminol.

[33] Markert K, Du Chesne A, Wunderlich G (1974) Tödlicher Mundschuß durch Viehbetäubungsapparat – zugleich ein Beitrag zur gerichtsmedizinischen Relevanz der Bolzenschußverletzungen. Kriminal Forens Wiss 17:107–124

[34] Maurer H (1961). Verletzungen durch Schußapparate. Beitr Gerichtl Med.

[35] Nadjem H, Pollak S (1993). Kombinierte Suizide unter Verwendung von Viehbetäubungsapparaten. Med Sach.

[36] Nadjem H, Pollak S (1999). Wound entry findings of animal anesthesia guns without smoke outlets. Arch Kriminol.

[37] Nikolić S, Zirković V, Juković F (2011). Planned complex occupation-related suicide by captive-bolt gunshot and hanging. J Forensic Sci.

[38] Ondruschka B, Heil K, Schulz S, Dreßler J, Morgenthal S (2018). Suicide or homicide? Forensic evaluation of fatal bolt gun injuries. Rechtsmedizin.

[39] Pircher R, Geisenberger D, Große Perdekamp M, Neukamm M, Pollak S, Schmidt U, Thierauf-Emberger A (2017). Suicide with two makes of captive-bolt guns (livestock stunners) fired simultaneously to the forehead. Int J Legal Med.

[40] Pollak S (1977). Morphology of injuries by ‘humane killer’ (livestock stunner). Z Rechtsmed.

[41] Pollak S, Maurer H (1987). On the clinical importance of the imprimatum in injuries from slaughterer’s gun (livestock narcotic device, “humane killer”). Acta Chir Austriaca.

[42] Pollak S, Reiter C (1981). ‘Bolt projectiles’ discharged from modified humane killers. Z Rechtsmed.

[43] Pollak S, Rothschild MA (2004). Gunshot injuries as a topic of medicolegal research in the German-speaking countries from the beginning of the 20th century up to the present time. Forensic Sci Int.

[44] Pollak S, Saukko P (2003) Atlas of forensic medicine, CD-ROM, Elsevier, Amsterdam, Fig 7.4.01–7.4.09

[45] Pollak S, Saukko P, Siegel JA, Saukko P (2013). Humane killing tools. Encyclopedia of forensic science.

[46] Prokop O, Göhler W (1976). Forensische Medizin 3rd ed.

[47] Rabl W, Sigrist T (1992). Suicide with a “humane killer” – skull fractures far removed from the penetration zone. Rechtsmedizin.

[48] Reitberger L (1951). Tierschußapparat – eine seltene Mordwaffe. Kriminalistik.

[49] Rothschild MA (2011) Conventional forensic medicine. In: Kneubuehl BP, Coupland RM, Rothschild MA, Thali MJ: Wound ballistics. Basics and applications. Springer, Berlin Heidelberg, pp 253–285

[50] Schiermeyer M (1973). Suizid durch zweimaligen Bolzenschuß in den Kopf. Arch Kriminol.

[51] Schollmeyer W, Disse M (1961). Sechs Selbstmorde und ein Mord mittels Bolzenschußapparat. Arch Kriminol.

[52] Schyma C, Hagemeier L, Madea B (2009). Wundballistische Überlegungen zum Bolzenschuss. Rechtsmedizin.

[53] Simic M, Draskovic D, Stojilkovic G, Vukovic R, Budimlija ZM (2007). The characteristics of head wounds inflicted by “humane killer” (captive-bolt gun) – a 15-year study. J Forensic Sci.

[54] Simon G (1958). Suicide, Tötungen und Verletzungen durch Viehschußapparate. Arch Psychiatr Nervenkr.

[55] Taschen B, Kühn E (1951). Selbstmorde und Mord durch Bolzenschußapparate. Kriminalistik.

[56] Tordrup PJ, Kjeldsen SR (1994). Accidental injuries from captive-bolt guns (slaughterer’s gun). Injury.

[57] Tovo S (1956). Un nuovo caso di suicidio con pistola da macellazione. Minerva Med.

[58] Ventura F, Blasi C, Celesti R (2002). Suicide with the latest type of slaughterer’s gun. Am J Forensic Med Pathol.

[59] Viel G, Schröder AS, Püschel K, Braun C (2009). Planned complex suicide by penetrating captive-bolt gunshot and hanging: case study and review of the literature. Forensic Sci Int.

[60] Wirth I, Markert K (1983) Halsmarkdurchtrennung durch Mundschuß mit Tierbetäubungsapparat – Kasuistischer Beitrag mit Literaturübersicht. Kriminal Forens Wiss 51/52:132–137

[61] Wirth I, Markert K, Strauch H (1983). Unusual suicides by livestock narcotic devices. Z Rechtsmed.

[62] Wolff F, Laufer M (1965). Über Bolzenschußverletzungen. Dtsch. Z Ges Gerichtl Med.

